# Combined Percutaneous and Endoscopic Ureterolithotripsy of Calcified Double J Stent

**DOI:** 10.7759/cureus.61431

**Published:** 2024-05-31

**Authors:** Luisa F de Arruda, Guilherme C Gonzales, João Henrique G Rodrigues, Ana Clara N Spessoto, Pedro Francisco F de Arruda

**Affiliations:** 1 Medicine, Medical School of Ribeirão Preto, Ribeirão Preto, BRA; 2 Urology, Faculty of Medicine of São José do Rio Preto (FAMERP), São José do Rio Preto, BRA; 3 Medicine, Medical School of Catanduva, Catanduva, BRA

**Keywords:** calcified ureteral stent, urology, ureterolithotripsy, endourology, double j stent, calculosis

## Abstract

In urological practice, the routine procedure of placing a double J stent aims to facilitate drainage of the upper urinary system. Despite its temporary nature and the necessity for timely removal, approximately 12% of these stents are retained in patients for extended durations due to various reasons. Forgotten ureteral stents can lead to complications that increase the morbidity and mortality of patients. This report discusses a case of the double J stent that became calcified due to prolonged use and needed to be removed in a combined procedure.

## Introduction

The placement of a double J stent has become routine in urological practice [1,2]. This procedure is indicated for the treatment of obstruction of the ureters due to ureteral calculosis, infection, external obstructions, compression, or malignant causes and to promote ureteral healing [1,2]. The main objective of the stent is drainage of the upper urinary system. Use is temporary and the stent needs to be removed prior to the end of its useful life, which depends on the brand of stent employed [1,2]. However, an estimated 12% of double J stents remain in the patient for a longer period for different reasons, such as loss of follow-up and even medical error [1,2]. 

Forgotten ureteral stents can lead to complications, such as calcifications, local infection, sepsis, and post-renal kidney failure, which increase the morbidity and mortality of patients. Severe calcification makes endoscopic removal difficult or even impossible. Indeed, removing a strongly encrusted stent from the urinary system constitutes a challenge for urologists [1,3]. 

Here, we describe the case of a 41-year-old male with a double J stent that became calcified due to prolonged use and needed to be removed in a combined procedure (percutaneous and endoscopic ureterolithotripsy).

## Case presentation

A 41-year-old male patient visited the emergency ward of a university hospital with a complaint of a strong pain in the left flank that began one day earlier, irradiating to the left iliac fossa and genital region. His physical examination revealed muscle tensing on abdominal palpation and possible left costovertebral angle tenderness. The patient's computed tomography revealed obstructive calculus in the left renal pelvis. A double J stent was implanted on the left side in November 2018. 

**Figure 1:**
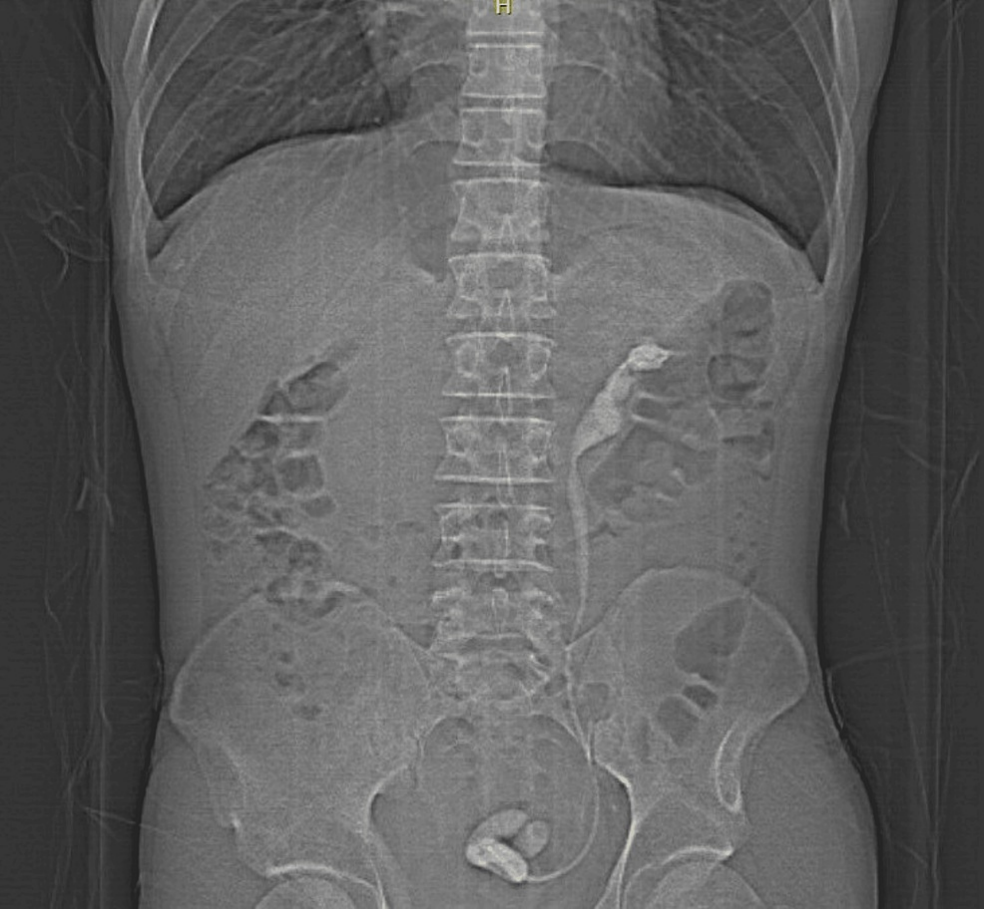
Abdominal X-ray image revealing calcification throughout the entire trajectory of double J stent (renal pelvis, ureter, and urinary bladder).

The patient returned after approximately four years (September 2022) reporting being asymptomatic during the period. However, the patient presented with dysuria, hematuria, and a strong pain in the left quadrant of the abdomen without irradiation that began one day prior to the second admission to the hospital. An abdominal radiograph was performed due to the immediate availability of the examination (Figure 1).

**Figure 2:**
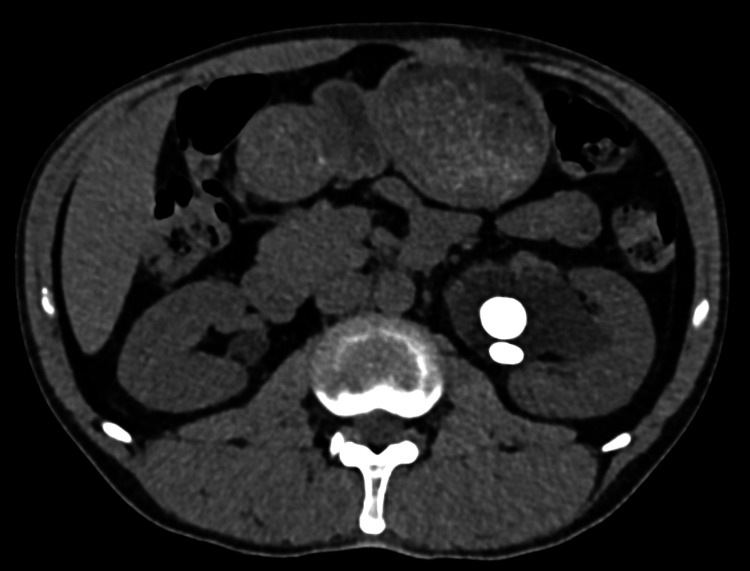
Computed tomography of abdomen showing calcification in left renal pelvis.

Computed tomography revealed an oval calculus in the left renal pelvis, the double J stent with calcifications, and calculi deposited on the bladder floor (Figure 2). 

**Figure 3:**
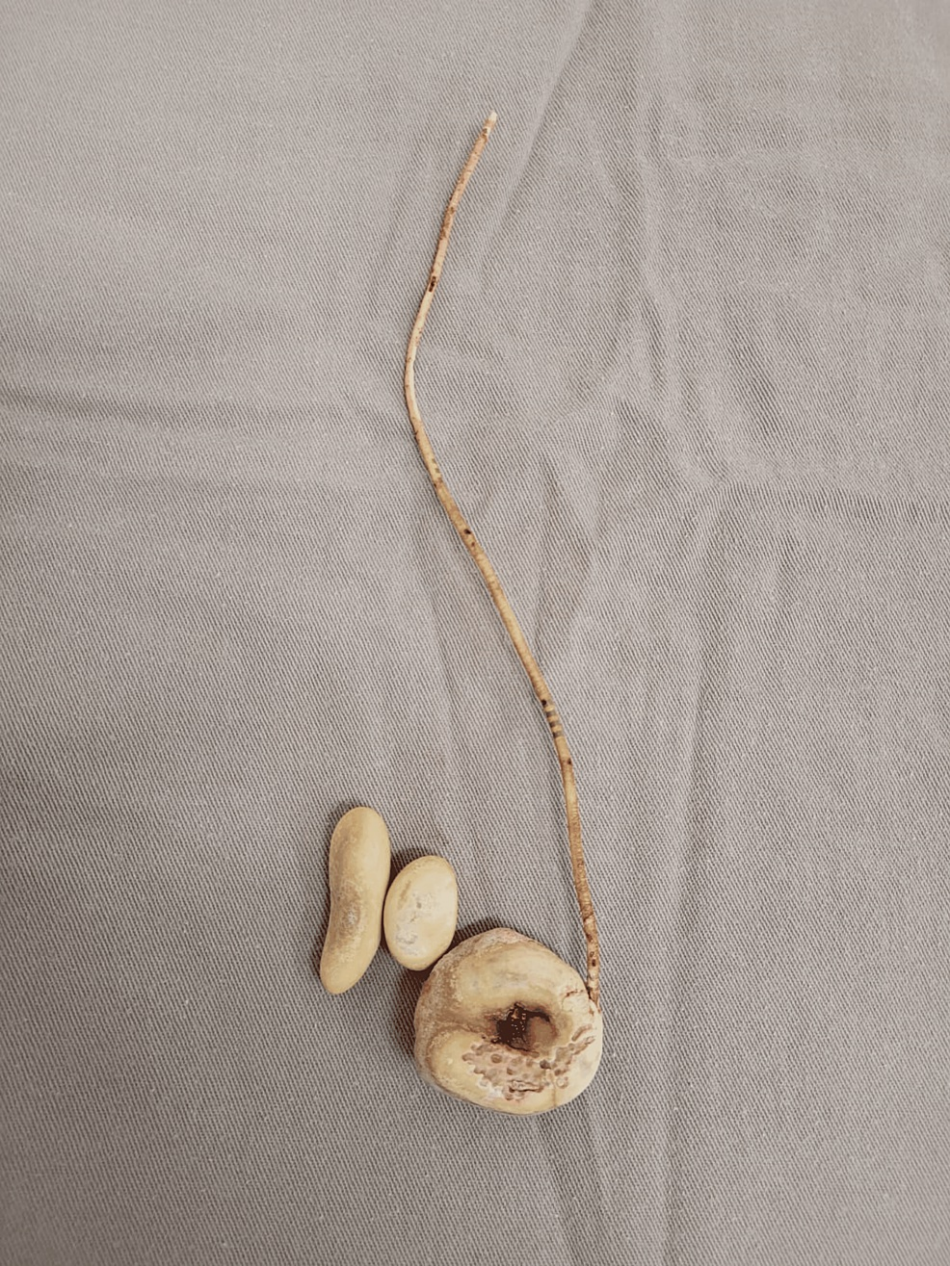
Products of cystostomy (first approach): calculi and calcified double J stent.

Considering the findings, the urology team planned surgery in two steps. The first was cystolithotomy, with the removal of calculi and a small segment of the double J stent (Figure 3). With no complications, the patient was discharged and instructed to return after one month. 

Upon return, the patient was submitted to percutaneous nephrolithotomy (Valdivia position) combined with laser ureterolithotripsy. This combination ensured successful fragmentation of the ureteral calcification and renal calcification. 

**Figure 4:**
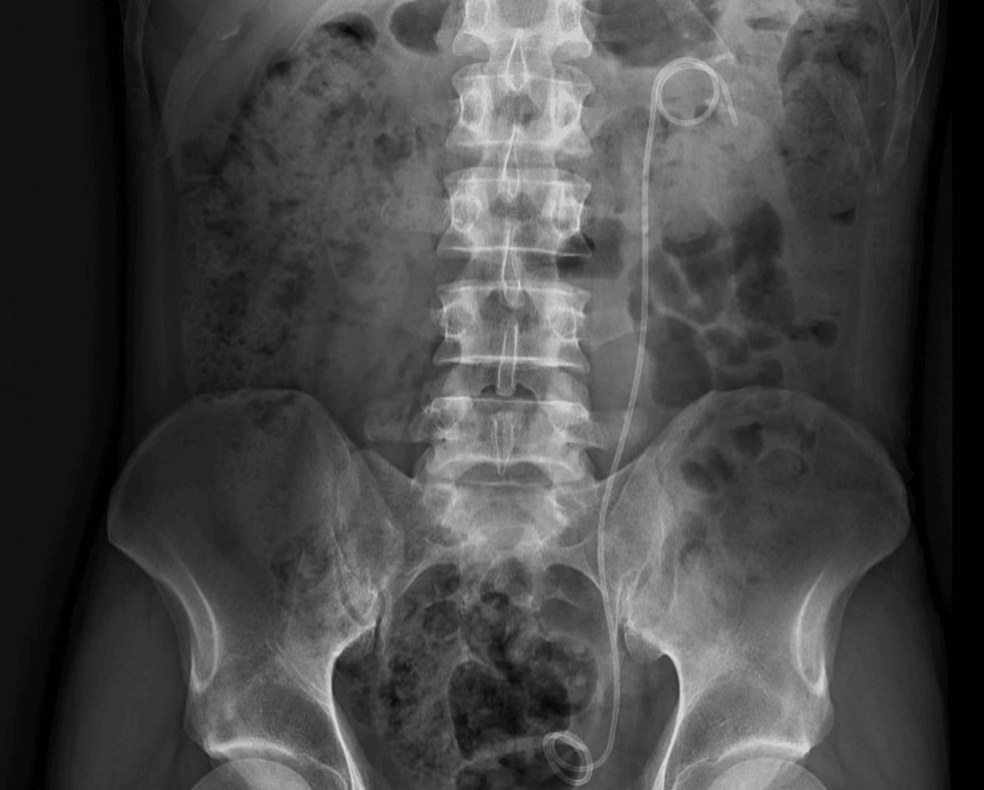
X-ray image showing new double J stent implanted on left side; ureter and kidney free of calculi.

Due to the substantial manipulation in the ureter, a new double J stent was installed to ensure drainage and healing of the ureter (Figure 4 shows the abdominal X-ray after the procedure). Upon discharge, the patient and family received counseling on the need for the definitive removal of the stent after one month. 

## Discussion

Encrustation of the double J stent is an event that can be avoided with the exchange or definitive treatment of the base cause of obstruction in a timely manner. However, complications related to the formation of calculi and encrustation of the stent due to the prolonged presence of the stent can lead to serious kidney damage and increased risk upon its removal. 

Most authors report prolonged presence to be the main cause of stent encrustation. Colonization by bacteria, urinary tract infection, pregnancy, and chemotherapy are also commonly described. El-Faqih et al. found that encrustation occurred in 9.2% of stents that remained in the kidney for less than six weeks, 47.5% of those that remained for six to 12 weeks, and 76.3% of those that remained for more than 12 weeks, considering double J stents installed due to calculous obstruction [3]. 

Most devices can be removed by endoscopic or percutaneous surgery with the support of an energy source, such as laser. For the patient described in this study, a combined method was used due to the formation of new calculi, substantial calcification, and pain. This combined approach aimed to diminish the need for further surgical interventions and nephrectomy and avoid the functional loss of the renal parenchyma. 

Forgotten double J stents and associated complications are common in the literature, especially in public health care services and in individuals with a lower income due to the difficulty in following up with these patients, who are unaware of the severity of the condition [4]. Such cases are complex and require multiple urological procedures to remove the stent and calculi, including shock wave lithotripsy, percutaneous surgery, ureteral and renal lithotripsy, nephrectomy [5], or a combined approach, as performed in the present case, which ensured a favorable outcome and the preservation of renal function.

As a means to prevent the prolonged retention of such devices beyond their recommended duration and thereby reduce their calcifications, computerized systems can be developed to automatically list patients with double J stents when the procedure is performed and documented in the electronic medical record. Once the list is generated, electronic medical record software can issue an alert to the medical team when such patients are nearing the expiration date of the double J catheter.

## Conclusions

The function of the double J stent is well-established in the literature. However, like other procedures and devices, the use of this stent has associated risks, some of which are predictable and others are not. In the present case, the loss of the patient to follow-up and the prolongation of the stent in the kidney beyond the necessary period led to complications that could have been more serious if not promptly treated by the urology team with the proposed combined method. 

Patients with a double J stent need to be monitored and followed up. After the placement of the stent, patients should be monitored to ensure the continuity of treatment and avoid the maintenance of the stent for a longer time than necessary. The urologist is responsible for the follow-up and removal of the stent in a timely manner, thus avoiding complications. In the present case, complete calcification of the double J stent occurred, the combined treatment of which is described little in the literature. The use of two procedures was of considerable importance, with a proper differential diagnosis, overall planning of the approach, and objective determination of the prognosis.
